# Supplementary material for: Dendritic Spine in Autism Genetics: Whole-Exome Sequencing Identifying De Novo Variant of CTTNBP2 in a Quad Family Affected by Autism Spectrum Disorder
Source: Children (Basel). 2022 Dec 30;10(1):80. doi: 10.3390/children10010080 (PMC9856540; doi:10.3390/children10010080)
Supplement: Supplementary file 1 [file children-10-00080-s001.zip › children-2044403-supplementary/children-2044403-Supplementary/Supplementary Appendix-R1.pdf]

Supplementary Appendix

Supplementary Figures

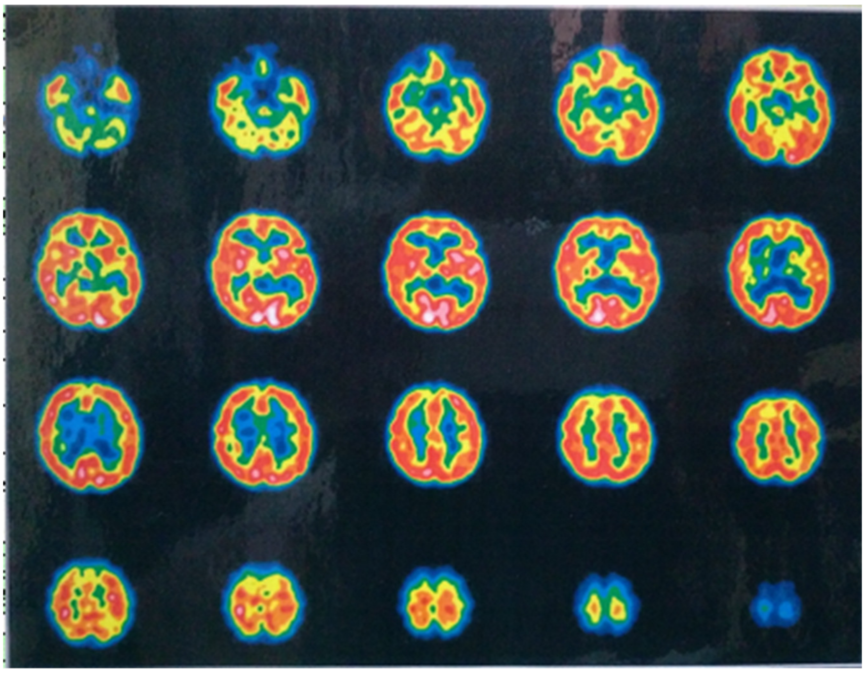

Supplementary Figure S1. Emission Computed Tomography showed focal perfusion and dysfunction.

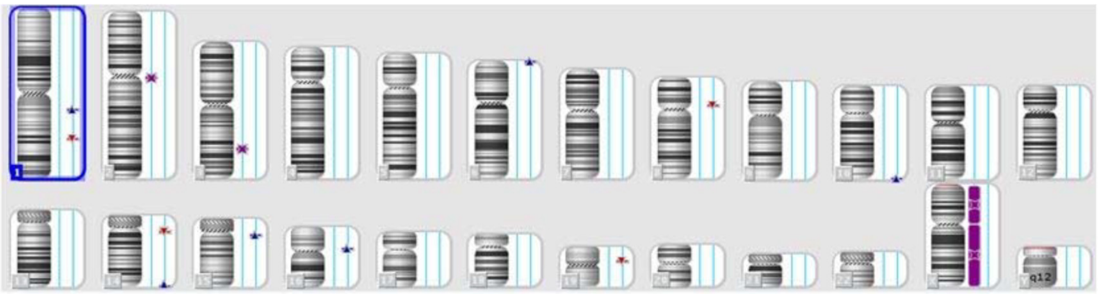

Chr...	▲ Type	▼ CN State	Size (kbp)	Cytoband Start	Cytoband End	Genes
1	▲ Gain	3.00	1,076	q21.2	q21.2	NBPF15, NBPF16, PPIAL4E, PPIAL4F, PPIAL4D, LOC645166, LOC388692, FCGR1C, PPIAL4A, PPIAL4C, ...
6	▲ Gain	3.00	100	p25.3	p25.3	DUSP22
10	▲ Gain	3.00	144	q26.3	q26.3	MTG1, SPRN, LOC619207, CYP2E1, SYCE1
14	▲ Gain	3.00	844	q32.33	q32.33	ELK2AP, KIAA0125, ADAM6, LINC00226
15	▲ Gain	3.00	351	q11.2	q11.2	PWRN1
16	▲ Gain	3.00	1,348	p11.2	p11.2	TP53TG3B, TP53TG3, TP53TG3C, SLC6A10P, LOC390705, RNU6-76P
1	▼ Loss	1.00	155	q31.2	q31.2	
8	▼ Loss	1.00	157	p11.22	p11.22	ADAM5, ADAM3A
14	▼ Loss	1.00	55	q11.2	q11.2	
14	▼ Loss	1.00	69	q11.2	q11.2	
19	▼ Loss	1.00	100	p12	p12	ZNF826P
2	LOH		3,915	q11.1	q11.2	FAM95A, ANKRD20A8P, LOC442028, TEKT4, MAL, MRPS5, ZNF514, ZNF2, PROM2, KCNIP3, ...
3	LOH		3,041	q25.2	q25.31	ARHGEF26-AS1, ARHGEF26, DHX36, GPR149, MME, LOC100507537, PLCH1, C3orf33, SLC33A1, GMPS, ...
X	LOH		55,634	p22.33	p11.1	XG, GYG2, ARSD, ARSE, ARSH, ARSF, CXorf28, MXRA5, PRKX, LOC389906, ...
X	LOH		93,044	q11.1	q28	SPIN4, LOC92249, ARHGEF9, MIR1468, AMER1, ASB12, MTMR8, ZC4H2, ZC3H12B, LAS1L, ...

Supplementary Figure S2. Chromosome Microarray Report of proband. ▲ in

blue means copy number gain, ▼ in red means copy number loss, LOH, Loss of Heterozygosity;

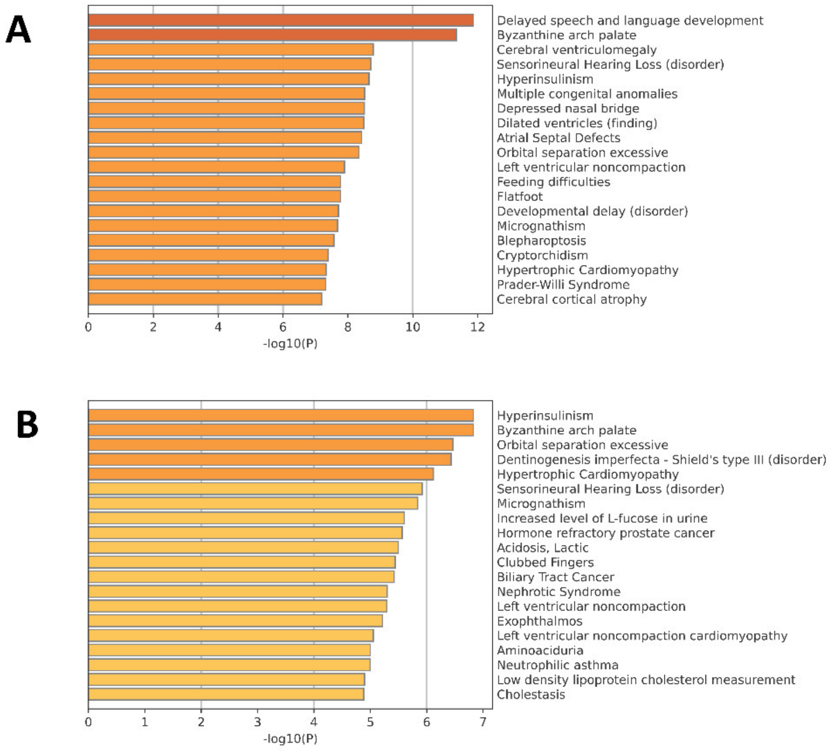

Supplementary Figure S3. Genes Enriched analysis. After initially filtering the public database for common variants and synonymous variants, a total of 541 variants were obtained from 248 genes, and after removing the SFARI genes, a total of 218 genes were obtained, the pathway enrichment analysis was performed using Metascape. A, all 248 genes were enriched to Delayed speech and language development and Byzanthine arch palate pathways; B, the 218 genes after removal of SFARI genes were enriched to Hyperinsulinism and Byzanthine arch palate pathways.
